# Editorial: Quality and quantity in research assessment: examining the merits of metrics, volume II

**DOI:** 10.3389/frma.2024.1400009

**Published:** 2024-03-26

**Authors:** Maziar Montazerian, Najmeh Shaghaei, Thea Marie Drachen, Bertil Fabricius Dorch

**Affiliations:** ^1^Department of Materials Science and Engineering, The Pennsylvania State University, University Park, PA, United States; ^2^University Library of Southern Denmark, Odense, Denmark; ^3^Department of Physics, Chemistry, and Pharmacy, Odense, Denmark

**Keywords:** assessment, quality, h-index, citation, research

Building upon the success of the first volume, the second volume of the Research Topic on “*Quality and quantity in research assessment: examining the merits of metrics*” aims to delve into the complex nature of metrics, their implications, and their role in the scholarly landscape.

In this era of data-driven decision-making, metrics have become integral when assessing the impact, visibility, and significance of research outputs. However, the use of metrics is not without its complexities and controversies (Leydesdorff et al., 2016). As the editors of this Research Topic, we have aimed to showcase a collection of articles that examine these nuances, presenting diverse perspectives and thought-provoking insights.

The articles published in this volume provide viewpoints from scholars, researchers, and practitioners across various disciplines. From bibliometric analyses to case studies, each contribution adds a layer to our understanding of how metrics shape the scholarly ecosystem (Leydesdorff et al., 2016; Waltman, 2016; Montazerian and Dorch, 2022).

One of the recurring subjects in these articles is the need for a balanced approach to research assessment. While metrics can provide quantifiable measures of productivity and impact, they often fall short in capturing the full spectrum of scholarly contributions. As such, many authors around the world lean toward developing more holistic evaluation frameworks that consider qualitative aspects alongside quantitative metrics. In this respect, the Coalition for Advancing Research Assessment (CoARA) comes along to ensure that research quality remains the core principle and enable recognition of the diverse practices and activities that maximize the quality of research.

Through checking out this Research Topic, readers will encounter discussions on the challenges posed by prevailing metric-based evaluation systems. Issues such as overemphasis on citation counts, incentivizing short-term results, discouraging risk-taking and innovation, field and discipline biases, manipulation research, etc. that prompt critical reflections on the unintended consequences of over-reliance on metrics.

However, these challenges need innovation and improvement. Several articles propose alternative metrics, novel methodologies, and best practices for responsible metric use (Montazerian et al., 2019, 2020; Montazerian and Dorch, 2022). By harnessing the power of metrics while remaining mindful of their limitations, the scholarly community can pave the way for a more robust and equitable research evaluation landscape.

The article “*Dark citations to federal resources and their contribution to the public health literature*” (Keralis et al.) examines the prevalence of “dark citations” - citations of information products outside of traditional peer-reviewed journal articles - in biomedical and public health literature, focusing on U.S. government sources. Dark citations are unlinked to indexed identifiers, often from government guidelines and informational products not systematically indexed. Surveying PubMed, the study identifies 96,690 dark citations from U.S. government domains, with 94% from federal agencies. COVID-19 publications contributed significantly. Notably, the U.S. Department of Health and Human Services (HHS) and its sub-agencies, particularly the Centers for Disease Control and Prevention (CDC) and the National Center for Health Statistics (NCHS), featured prominently. These findings suggest the growing importance of non-traditional citations from government sources, indicating a need for their inclusion in bibliometric analyses to accurately measure research impact.

The article “*Development and preliminary validation of an open access, open data, and open outreach indicator*” (Vlachos et al.) introduces the OADO (Open Access, Open Data, and Open Outreach) indicator, designed to assess researchers' openness. The OADO comprises two factors: the research factor, gauging the presence of Open Access (OA) articles and Open Data (OD) in research, and the communication factor, measuring Open Outreach (OO) in public engagement activities. Developed for Elsevier's Research Information Management System (RIMS) Pure, but useable on any RIMS with information on open data, outreach and access, this indicator offers nuanced insights into researchers' openness within their discipline or department. Tested on 995 researchers from the University of Southern Denmark, the Weighted-OADO highlights variations in openness across faculties. The OADO presents a promising, citation-database-independent tool for evaluating and fostering open science practices, offering actionable insights for institutions to support and recognize researchers' efforts toward openness.

The article “*Research metrics for health science schools: a conceptual exploration and proposal*” by Gemechu et al. proposes a much-needed standardized framework for measuring the return on investment (ROI) in public health research. Highlighting the absence of universally accepted metrics, the authors present a comprehensive model categorizing metrics into the research lifecycle's four stages: Input, Process, Output, and Outcome/Impact. The article reviews existing frameworks, noting their strengths and limitations, and emphasizes the importance of standardized terminology and data collection methods. It acknowledges challenges such as diverse stakeholder interests and resource limitations. Overall, this work serves as a foundational guide for institutions seeking to develop robust research metrics systems. It encourages dialogue on standardized research measures across health science schools, aiming to improve the effectiveness and impact of public health research.

Furthermore, Olejniczak et al. discuss the pivotal role of bibliometrics in guiding decisions within research universities, emphasizing the need for a nuanced approach across diverse disciplines. It highlights the limitations of current ranking schemes, often biased toward journal articles, neglecting varied publication modalities like conference proceedings and books. The article advocates for a comprehensive understanding of disciplinary publishing practices, stressing the importance of discipline-specific normalization techniques. It proposes assessing publishing outputs based on medians rather than means to counter skewed distributions, and advocates for accounting for disciplinary rhythms and career stages. The note ultimately calls for a balanced approach, combining quantitative bibliometric data with qualitative expert assessment to ensure accurate and fair evaluations.

As we analyze this collection of articles and numerous others, we witness a continuously evolving landscape of research assessment. To enhance these efforts, it is necessary to maintain an open and inclusive dialogue. This Research Topic serves as a testament to the vibrant discussions and diverse perspectives that drive progress in this field. We hope that the articles presented here will inspire further exploration, spark new ideas, and foster collaborations aimed at refining how we evaluate research in the twenty first century.

## Author contributions

MM: Writing—original draft, Writing—review & editing. NS: Writing—review & editing. TD: Writing—review & editing. BD: Writing—review & editing.
